# Conceptions of sleep experience: a layman perspective

**DOI:** 10.1186/s13104-018-3584-2

**Published:** 2018-07-18

**Authors:** Maaike S. Goelema, Renske de Bruijn, Sebastiaan Overeem, Els Møst, Reinder Haakma, Panos Markopoulos

**Affiliations:** 1 Philips Group Innovation Research, High Tech Campus 34, 5600 MB Eindhoven, The Netherlands; 2 Department of Industrial Design, Eindhoven University of Technology, Den Rondom 70, 5612 AZ Eindhoven, The Netherlands; 3 Department of Human Technology Interaction, Eindhoven University of Technology, Eindhoven, The Netherlands; 4 Sleep Medicine Center, Kempenhaeghe, Heeze, The Netherlands

**Keywords:** Perceived sleep quality, Sleep behavior, Health monitoring, Phenomenology

## Abstract

**Objective:**

To date, there is little information on how lay people understand and discuss sleep in the context of daily life. Efforts to conceptualize sleep quality have been largely driven by clinical considerations of sleep disorders. As such, they are not necessarily of how normal sleepers without clinical expertise conceptualize sleep quality. A phenomenological approach was taken to understand the essence of the sleep experience and the concepts held by lay people without sleep disorders. A sentence completion questionnaire was developed and administered to a quota sample of 64 respondents who were selected aiming for sufficient representation of different gender, ages, and education levels.

**Results:**

Significant sentences and meaningful units were derived inductively, resulting in a classification of nine categories. The major facets of sleep experience of lay people were ‘daytime functioning’, ‘interruptions during the night’ and ‘before bed state’. This implies that the experienced sleep quality is not only depending on the progress of the night. These results can guide future research to provide suitable psychometric measures for normal sleepers, as well as the design of sleep data visualization applications in the context of health self-monitoring.

**Electronic supplementary material:**

The online version of this article (10.1186/s13104-018-3584-2) contains supplementary material, which is available to authorized users.

## Introduction

In recent years, there is a growing trend towards self-monitoring of sleep quality using commodity electronics, typically based on self-report or activity level measurements [1]. While such technologies are not yet meant as a tool to support clinical practice, the need arises to represent sleep quality in terms that are understood by people and are meaningful with respect to how they experience sleep [2, 3]. In addition, since sleep is related to a variety of health outcomes [4–6], understanding how lay people describe and understand sleep is important as addressing certain sleep issues may improve other health outcomes as well.

Defining and operationalizing sleep quality is challenging and a broadly accepted definition is still lacking. Traditionally clinical research has sought to define and operationalize sleep qualities by developing purpose specific questionnaires that are based on a clinical understanding of sleep disorders. Such questionnaires are not necessarily reflective of how lay people without sleep disorders conceptualize sleep and think about their own sleep experience. Krystal and Edinger [7] proposed to measure sleep quality based on the Likert-style rating of sleep quality of the previous night without attempting to further detail or conceptualize what sleep quality should be assumed to refer to.

Research regarding the sleep experience of normal sleepers is limited [8–10]. Harvey et al. [8] conducted structured interviews which helped them identify the following factors as the most important for judging subjective sleep quality the morning after: whether you got enough sleep, how tired you feel throughout the day, how rested you feel when waking up, feeling restored on waking and feeling alert throughout the day. Buysse et al. [11] observed that subjective sleep depth falls along the same dimension as overall sleep satisfaction. A broader consideration may be needed as to what the general sleep experience is for normal sleepers, starting from what someone needs to go to sleep, to how a person awakes the best, to improve our understanding of what is a good sleep experience and subsequently sleep quality.

Since there is little information on how lay people understand and discuss sleep in the context of daily life, the aim of the current paper is to provide insight into the sleep experience of lay people. A phenomenological research approach is taken that aims to capture the essence of an experience common to a group of people, bracketing out prior conceptions of this experience on behalf of the researcher.

## Main text

### Methods

#### Participants

Ethical approval was received from the Institutional Ethical Review Board of Eindhoven University of Technology. Participants were recruited through a database maintained at the University and approached by email. Participants could interact with the researchers through a website. The whole study was conducted online. A quota sampling approach was applied in order to ensure that the following subgroups were sufficiently represented in the final sample:

- Age: young (≤ 49 years)/old (≥ 50 years).
- Sex: female/male.
- Education: high (≥ bachelor)/low (< bachelor).
- Pittsburgh Sleep Quality Index (PSQI) [12]: good (≤ 5)/poor (> 5).

In each of the combination of these groups, we had at least 4 participants, which made the total number of participants for the analysis of this study 64 (2 age * 2 sex * 2 education * 2 PSQI * 4 = 64 participants). Persons with a formally diagnosed sleep disorder were excluded from participation.

However, we did not actively screen the sample for signs of—undiagnosed-sleep disorder, as the wider community population also consists of people who experience sleep problems and people who do not.

#### Measures

Sentence stem completion questionnaires [13] were used to survey participants’ conceptions of sleep experience. Stem completion is a projective data collection technique which was originally developed as part of an intelligence test. Initially, a pool of 62 Dutch sentence stems was derived based on examination of existing subjective sleep quality inventories [12, 14–17]. A pilot trial of the 62-item stem completion questionnaire was carried out with 10 individuals, recruited through convenience sampling amongst colleagues in the research groups of the authors. The responses were analyzed for word frequency and comparisons were made between responses depending on whether a participant indicated to be sleeping well, poorly, or neutral. Removing stems that did not seem to provide new information left 30 sentence stems over, resulting in the Sleep Sentence Completion Questionnaire (SSCQ). Translations of the items of the questionnaire can be found in Additional file 1.

#### Procedure

After informed consent we checked that participants were native Dutch speakers, to ensure adequate comprehension of the stems and the ability to express themselves adequately. Filling in the survey took 15′ min. Demographic information, educational level, the Dutch version of the Pittsburgh Sleep Quality Index (PSQI) [12] and SSCQ were assessed. The PSQI is a validated questionnaire, for the assessment of sleep quality, with a Dutch translation available (Sillis and Cluydts, unpublished data of ‘personal communication’). Objective sleep measurements were not included as it was not relevant for our research question whether people had a good or poor night’s of sleep. In addition, most sleep questionnaires are not necessarily reflective of how lay people perceive their sleep. Finally, previous studies observed low to medium associations between objective sleep measurements and subjective sleep quality [18–21].

#### Data analysis

A phenomenological data analysis approach was adopted. Phenomenology is a qualitative research approach which aims at describing the common meaning of lived experiences for several individuals, reducing the experiences reported by individuals to a central meaning, or the ‘essence’ of the experience [22].

Completed questionnaires were read one by one in their entirety and analyzed to identify the most meaningful statements per participant (in total 64 participants). No fixed number was set, but we aimed for selecting approximately 1–5 meaningful statements per participant in order to reduce the data set. Selected meaningful statements for the full set of participants were clustered into themes, now representing the whole participant population. This resulted in a set of 206 significant statements, which were clustered into eight themes, based on the frequency of the statements and their similarities. Since the statements were already short in itself no software was used to analyze the statements.

To ensure the validity of the clustering, two coders performed independently a direct content analysis [23] where they classified all the statements (64 × 30 = 1920) along the eight themes. After 5% of the data, a comparison table was created to identify divergent classifications and interpretations. Based on this discussion, another category was added, namely: alarm clock. The remaining 1800 statements were classified by the two coders achieving a consistency of 0.81 Cohen’s kappa.

### Results

The characteristics of the study sample are displayed in Table 1. The average total sleep time of the study sample was ± 8 h.

**Table 1:** Participants characteristics

Characteristics	
Age, mean (SD)	47 (18.2)
Age range	18–79
Gender	32 (50% female)
PSQI, mean (SD)	5.9 (3.7)
PSQI range	1–16
Time to bed, mean (SD)	23:38:59 (1:13:42)
Wake up time, mean (SD)	7:31:41 (1:11:35)

*SD* standard deviation

The nine themes that resulted from the data analysis can be found in Table 2. The largest category in our analysis was ‘next day state’, characterized by the following statements: whether people felt tired the next day, whether they felt restored the next day, how refreshed or rested they felt when waking up or whether they felt energetic during the day.

**Table 2:** The outcome of the categorization of the 206 statements about sleep experiences

Themes	Description	Example	#statements
Next day state	Includes statements about the state of well-being directly after waking up and daytime functioning	*After a good night’s sleep: I feel fit and energized, and looking forward to the day*	51
Interruptions during the night	Contains statements about waking up through the night either induced by internal causes (toilet visits) or external causes (noise). Also includes the positive stated statements, such as sleeping through the night	*A good night’s sleep is: minimizing the number of awakenings during the night*	41
Before bed state	Involves statements about mental and physical well-being before going to bed	*What most affected my sleep was: stress*	39
Sleep characteristics	Contains the other sleep parameters such as sleep onset latency, deep sleep and sleep duration	*I feel best when: I have slept 8* *h*	18
Bedroom environment	This theme is about all the objects in the bedroom (for instance, bed, mattress, pillow etc.) and the light and temperature in the bedroom	*What my sleep interrupts is: too much light through the window*	13
Thoughts about sleep	Statements about how they perceive sleep and the function of sleep	*I experience my sleep: as important*	12
Routine	Contains statements about bedtime routines such as listening to music or drinking habits before bedtime	*Before bedtime: I would like to read a book*	11
Alarm clock	Statements that include either waking up with or without an alarm clock	*I cannot fall asleep without: first setting the alarm clock*	5
Other	This theme contains all the other statements which do not belong to the other themes	*My sleep is poor when: I dream a lot*	16

Statements about sleeping through the night and waking up during the night were included in the second category: interruptions. The interruptions category can be divided into two parts, one part where internal reasons were given for the interruptions during the night (waking up in general and toilet visits) and a second part where external reasons were given for not sleeping through the night (noise and waking up by others).

The third largest category ‘before bed state’ contained concepts about the mental status before going to bed and the body state in which subjects entered the bed. For instance, ‘I sleep well when I am not worried or stressed before bedtime’.

190 statements of the 206 statements were accommodated under the 8 categories. Statements that were not included in the 8 categories but were allocated in the category ‘other’ concerned: dreams/nightmares (4), regular bedtimes (2), bad food (3) and some few single statements (7). However, after the validity of the coding, the category ‘other’ came out quite large as statements about sleeping posture, sleep rhythm and sleeping on time could not be designated under the other categories but were mentioned quite often.

### Discussion

In our study, daytime functioning was one of the most important factors for describing the sleep experience. In other words, the energy level during the day, how tired or refreshed they felt when waking up, were frequently described when answering the SSCQ. This suggests that daytime functioning is more important for people to judge their sleep experience than the actual night itself. Still, wake time during the night cannot be ignored, as this also formed a large category in our analysis. In the study of Ramlee et al. [9] wake time during the night was not a major factor when judging sleep quality. Compared to the study of Harvey et al. [8], our participants indicated that their state of mind before bedtime is an import factor for the sleep experience. State of mind, such as stress, has been correlated with subjective sleep quality [24–26]. The categories are linked though, as stress before going to bed can cause a longer sleep onset latency, more awakenings during the night and therefore a shorter sleep duration [27, 28]. As a consequence, one may feel tired during the day. Overall our study gave insight in the whole sleep experience of lay people whereas (in general) abovementioned studies only considered judgements of participants on a good and poor night’s of sleep.

When comparing the PSQI with our categorization we saw one remarkable thing. The major category daytime functioning did not match with the questions of the PSQI, in particular, because of the different wording of the questions of the PSQI. The PSQI questions relate more to the extremes of daytime functioning, for instance: ‘During the past month, how often have you had trouble staying awake while driving, eating meals, or engaging in social activity?’. Probably, most people do experience some degree of dysfunction during the day because of tiredness or not feeling refreshed, however, they do not experience problems with staying awake.

As the perception of sleep is not always related to the actual sleep, this may explain the low correlations between objective and subjective sleep measurements often found [19, 29–31]. For sleep monitoring devices or sleep coaching apps this is important to consider, as sometimes the data contrasts with what people feel. Additionally, this may implicate that the data shown by a device should be interpreted with caution.

This study was conducted with a balanced study sample. A phenomenological approach was used to analyze the qualitative data to capture the participant perspective and what they report as their sleep experience. The study results are likely generalizable to the broader community sample, which also contains a proportion of people with sleep problems, or undiagnosed sleep disorders. A suggestion for future research would be to develop a new validated sleep questionnaire that covers the whole range of the sleep experience to gain a better understanding of the subjective sleep quality.

### Conclusion

Our results imply that the experienced sleep quality is not only depending on the progress of the night. The sleep quality definition should, from a subjective point of view, be elaborated with other factors, such as stress/well-being levels, feeling of being rested and functioning during the day. These results can guide future research to provide suitable psychometric measures for normal sleepers, since some parts of the sleep experience of lay people are not addressed in the sleep quality questionnaires at the moment. In addition, with the emerging trend of consumer activity trackers above results could provide guidance in the design of the feedback towards the users, e.g. including educational information about sleep relaxation exercises or a tab where users can indicate their level of stress before going to bed. This will contribute to an insight in the overall sleep experience of the user.

## Limitations

Several limitations of our study should be acknowledged. While the use of a sentence completion questionnaire allowed an understanding of the intuitive sleep concepts of lay people, the questions of the SSCQ were guided questions as they asked about specific facets of sleep. More open questions may have yielded the risk that not every aspect of sleep was covered, as the pitfall emerges of repeating oneself. With the SSCQ we were able to capture the whole picture of participants’ perspectives about sleep. We tried to reduce the experimenter bias as much as possible through calculating the interrater reliability. Although several age groups were included in this study we cannot rule out that we have corrected for all the factors that impact sleep, such as unemployment, medication and chronic pain. Finally, since our study sample consisted of Dutch participant the results may not culturally generalizable to other countries.

## Additional file

**Additional file 1.** Sleep Sentence Completion Questionnaire. English translation of the Sleep Sentence Completion Questionnaire.

## Abbreviations

SSCQ: Sleep Sentence Completion Questionnaire
PSQI: Pittsburgh Sleep Quality Index
