# Supplementary material for: Conceptions of sleep experience: a layman perspective
Source: BMC Res Notes. 2018 Jul 18;11:494. doi: 10.1186/s13104-018-3584-2 (PMC6052564; doi:10.1186/s13104-018-3584-2)
Supplement: Supplementary file 1 — Additional file 1. Sleep Sentence Completion Questionnaire. English translation of the Sleep Sentence Completion Questionnaire. [file 13104_2018_3584_MOESM1_ESM.docx]

**Additional file 1: Sleep Sentence Completion Questionnaire**

The questionnaire is presented below in Dutch, together with its English translation.

| Normaal gezien voel ik me na het slapen .......... * | Normally after sleeping I feel………* |
| --- | --- |
| Na een nacht slecht slapen voel ik mij.......... * | After a bad night’s sleep I feel……… * |
| Ik ontwaak graag.......... * | I like to wake up………* |
| Een slechte nachtslaap is.......... * | A poor night’s sleep is.......... * |
| Ik slaap graag.......... * | I like to sleep.......... * |
| Ik slaap slecht wanneer.......... * | I sleep poorly when.......... * |
| Ik ervaar mijn slaap als.......... * | I experience my sleep as.......... * |
| Het belangrijkste aan slapen vind ik.......... * | I think the most important thing about sleeping is.......... * |
| Een goede nachtslaap is.......... * | A good night’s sleep is.......... * |
| Ik denk dat het moeilijk is om te slapen wanneer.......... * | I think it is difficult to sleep when.......... * |
| Na een nacht slecht slapen..........  * | After a poor night’s sleep..........  * |
| Ik voel me het best wanneer mijn slaap... * | I feel best when my sleep... * |
| Ik slaap goed wanneer ik..........ben * | I sleep well when I am.......... * |
| Hetgeen mijn slaap het meest beïnvloed.......... * | The thing that my sleep the most influences.......... * |
| Slaap is voor mij.......... * | Sleep is for me.......... * |
| Mijn slaap is slecht als.......... * | My sleep is poor when.......... * |
| Na een nacht goed slapen.......... * | After a good night’s sleep.......... * |
| Hetgeen mijn slaap onderbreekt is/zijn.......... * | What my sleep interrupts is/are.......... * |
| Voor het slapen gaan wil ik graag.......... * | Before bedtime I would like.......... * |
| Ik slaap slecht wanneer ik..........ben * | I sleep badly when I am..........* |
| Ik kan niet slapen zonder.......... * | I cannot sleep without.......... * |
| Mijn slaap vannacht was.......... * | My sleep tonight was.......... * |
| Mijn slaap is goed als.......... * | My sleep is good when.......... * |
| Ik ontwaak (vaak) gedurende de nacht  omdat.......... * | I wake up (often) during the night because......... * |
| Ik kan niet slapen zonder eerst.......... * | I cannot sleep without first.......... * |
| Na een nacht goed slapen voel ik mij.......... * | After a good night’s sleep I feel.......... * |
| Wat ik nodig heb om goed te slapen is/zijn.......... * | What I need to sleep good is/are.......... * |
| Wat me (soms) s'nachts wakker houdt is/zijn.......... * | The thing(s) that (sometimes) keep me awake at night is/are......... * |
| Ik slaap goed wanneer.......... * | I sleep well when.......... * |
| Mijn ideale slaap…. | My ideal sleep…. |
